# *Gordonia* sp.: a salt tolerant bacterial inoculant for growth promotion of pearl millet under saline soil conditions

**DOI:** 10.1007/s13205-013-0178-5

**Published:** 2013-11-02

**Authors:** Monika Kayasth, Varun Kumar, Rajesh Gera

**Affiliations:** 1 Department of Microbiology, CCS Haryana Agricultural University, Hisar, 125004 India; 2 Department of Biotechnology and Bioinformatics, Jaypee University of Information Technology, Waknaghat, 173234 Himachal Pradesh India

**Keywords:** Salt tolerant, Plant growth-promoting rhizobacterium, *nifH*, Pearl millet, *Gordonia* sp.

## Abstract

In the resent study, a diazotrophic bacterial isolate JPA2 having the ability to tolerate salinity (6 % NaCl) and plant growth-promoting features was isolated from rhizospheric soil of weed *Chenopodium murale* growing in saline soil of Pindara (EC 11.47 dS m^−1^), district Jind (Haryana). The nitrogen fixing ability of the isolate was confirmed by *nifH* gene amplification and acetylene reduction assay (38.9 nmol ethylene h^−1^ mg^−1^ protein). The potential of strain JPA2 to promote growth of pearl millet was investigated by inoculation experiment which showed significant increase in plant height (51.1, 39.9 and 28.8 %) and dry weight (55.9, 36.4 and 35.5 %) over uninoculated control plants at EC 0, 6, 8 dS m^−1^, respectively. The strain JPA2 was Gram +ve and identified as *Gordonia* sp. on the basis of partial 16S rRNA gene sequencing and biochemical characterization. It is concluded that salt tolerant diazotrophic *Gordonia* sp. can be considered as a beneficial microbe for agriculture in saline soils.

## Introduction

Saline soils are extensive in the plains of Haryana state (Choubey et al. 2009) particularly in arid and semi-arid regions with ~2,32,985 hectares of area is considered as salt affected. Soils containing excess amount of water soluble salts are called saline soils. The concentration of salt in the root-zone of soil goes so high that it badly affects growth of the most crop species. But some cereal species are well adapted to semi-arid and arid tropics, where salinity and drought are the major problems due to limited water supply. Among the cereals, pearl millet (*Pennisetum glaucum*) is the only major crop that has high level of tolerance to saline soils. It can be cultivated even in the most sandy infertile soils and saline environments where no other cereal crop can survive (Kumar et al. 2010). However, to survive in saline soils, the increased demand of nitrogen in pearl millet could be met by the exploitation of diazotrophic bacteria (Latake et al. 2009). Saline rhizosphere habitats are blessed with some free-living nitrogen fixing bacteria which siphon out appreciable amount of nitrogen from reservoirs and enrich the soil. So, it is important to exploit rhizobacteria which often play crucial role in increasing crop productivity of the plants they colonize due to their close proximity with the roots they inhabit. Plant growth-promoting rhizobacteria (PGPR) stimulate the plant growth by producing Indole-3-acetic acid (IAA) and synthesizing siderophores that chelate iron and make it available to plant root (Kumar and Gera 2013). The application of PGPR as crop inoculants for biofertilization would be an attractive alternative to decrease the use of chemical fertilizers which effect environmental pollution.

The genus *Gordonia*, a coryneform bacterial member, is widespread in nature and comprises over 33 species, with broader characteristics such as the ability to degrade hydrocarbons (Xue et al. 2003), toxic environmental pollutants, xenobiotics (Hernandez et al. 2001), and natural compounds that are not readily biodegradable. *Gordonia* sp. may play an important role during waste water treatment (Bendinger et al. 1992) and also used as PGPR for Zea mays (Hong et al. 2010). Even though the higher number of *Gordonia* species described, to the best of our knowledge, there are no in-depth studies on the occurrence of nitrogen fixing species within the genus in saline soils and their possible role in growth promotion of pearl millet. Therefore, in the present investigation, diazotrophic *Gordonia* sp. was isolated from rhizospheric soil of weed growing in saline soils and was further characterized for its PGP features. In addition, the inoculation effects of *Gordonia* sp. on the growth of pearl millet were evaluated in artificially created saline conditions under pot house experiment.

## Materials and methods

A salt affected barren field of Pindara village (latitude: 29°30′ N, longitude: 76°34′ E), district Jind, Haryana was chosen for sampling which harbors only few weeds such as *C. murale*. The weed rhizospheric soil sample was collected carefully by uprooting the root system and soil was placed in the sterile plastic bags for transport to the laboratory. Physiochemical analysis of soil properties, including pH, EC, organic carbon (Kalembasa and Jenkinson 1973) and total N (Bruel et al. 1946) was estimated. Three morphotypes were obtained on Jensen’s medium (Jensen 1951) and maintained on respective medium slants at 4 °C. To investigate their nitrogen fixing ability, the genomic DNA of cultures was subjected to *nifH* gene amplification using primers: *nifH* for (5′-TAY GGN AAR GGN GGHATY GGY ATC-3′) and *nifH* rev (5′-ATR TTR TTN GCN GCR TAV ABB GCC ATC AT-3′) (Sarita et al. 2007). The PCR reaction mixture (25 μl) contained 0.5 μl of dNTP mixture (10 mM), 0.5 μl of Taq DNA polymerase (3 U μl^−1^), 10× PCR assay buffer with 25 mM MgCl_2_ (2.5 μl), 2 μl of each primer (10 pmol each), 2 μl template DNA (0.1–0.14 μg/μl) and 15.5 μl sterile water. PCR protocol conditions were: initial denaturation at 95 °C for 4 min, 35 cycles of denaturation at 94 °C for 30 s, annealing at 54 °C for 1 min and extension at 72 °C for 1 min followed by a final extension at 72 °C for 5 min. The PCR was then set on hold at 4 °C. Amplified PCR product was separated by electrophoresis on 2 % agarose gel stained with ethidium bromide and photographed under UV illumination using Gel Documentation system (DNR Bio-Imaging Systems). The isolate having *nifH* gene designated as JPA2 was further examined for acetylene reduction according to the method of Bridage et al. (1982).

For the identification of the strain JPA2, its 16S rRNA region was amplified and partially sequenced (Bioserve, Hyderabad, India) by primer walking using five different internal primers (16SEQ2R, 16SEQ3F, INS16SREV, 16SEQ4R and 16SEQ4F). The resulting 16S rRNA gene sequence was compared with sequences in GenBank database using the BLAST-N algorithm to identify sequences with a high degree of similarity. Comparable reference sequences were downloaded in FASTA format from NCBI database (http://www.ncbi.nlm.nih.gov). All the sequences were aligned with ClustalW program and phylogenetic tree was constructed using MEGA4 software through neighbor-joining method. Bootstrap analysis was used to evaluate the tree topology of the neighbor-joining data with 1,000 resamplings. The partial 16S rRNA gene sequence determined in this study was deposited in the GenBank database (http://www.ncbi.nlm.nih.gov/Genbank/index.html) under the accession number JQ437540. Bacterial culture JPA2 was further characterized morphologically and subjected to a battery of biochemical tests such as nitrate reduction, hydrolysis of Aesculin and starch (Cowan and Steel 1965). Sugar utilization pattern was assayed according to Collins and Lyne (1984).

The ability of the strain JPA2 to produce IAA was detected using the rapid assay (Bric et al. 1991), ammonia production examined using Nessler’s reagent (Cappuccino and Sherman 1992) while siderophore production tested by CAS assay (Schwyn and Neilands 1987). Jensen’s medium modified to the saline concentration of 2–10 % NaCl was used to examine the capability of strain JPA2 to tolerate different concentrations of salinity.

 In vivo pot test for plant growth promotion was carried out at CCS Haryana Agricultural University, Hisar to evaluate the effect of salt tolerant PGPR isolate using pearl millet as test crop. Seeds of pearl millet (HHB 146: a salt tolerant variety) obtained from Bajra section, Agriculture college, CCS HAU, Hisar, were surface sterilized by exposing to 95 % ethanol and immersing in 0.2 % of HgCl_2_ solution for 3 min. The seeds were then subjected to five washings with sterile distilled water. Earthen pots with drainage hole were filled with 5 kg of soil and two salinity levels (EC 6, 8 dS m^−1^) were maintained through irrigation water by addition of different concentration of salts such as NaCl, CaCl_2_·2H_2_O, MgCl_2_ and MgSO_4_·7H_2_O while pots of EC 0 dS m^−1^ were irrigated with normal distilled water. Recommended dose of commercial fertilizer at the rate of 80 N and 40 P kg ha^−1^ was supplied to each pot through urea and single super phosphate, respectively. Nitrogen was applied in two splits, while full dose of phosphorus was applied as basal dose. Experiment was laid out in randomized block design with three replications per treatment. Four seeds were planted per pot at a uniform depth of 1.5 cm. For preparation of bacterial formulation, the strain JPA2 was cultured in LB medium and incubated on an orbital shaker at 150 rpm at 30 °C. 1 ml of this overnight grown bacterial suspension (10^6^–10^7^ cells ml^−1^, 0.5 O.D. at 540 nm) was applied to each pearl millet seed. However, in control, seeds were treated with sterile non-inoculated LB medium. The pots were kept in sunlight, irrigated and observed everyday for 45 days. On 46th day, plants were harvested carefully at vegetative stage. Shoot length was measured after harvesting and samples were dried in oven at 75 °C. After 4 days, shoot dry weight (g/pot) was calculated and statistical significance of the data was determined by ANOVA (Analysis of Variance) using MINITAB version 15.0 and Tukey’s tests (*P* = 0.05).

## Results and discussion

In the present investigation, a salt tolerant PGPR strain was isolated from rhizosphere of weed *Chenopodium murale* grown in saline fields of Pindara village. The rhizospheric soil sample has the following physiochemical properties: pH 6.24; 11.47 dS m^−1^ EC; 0.13 % organic carbon and 0.12 % total N. Three different morphotypes were selected on the basis of their colony color, shape and size on Jensen’s nitrogen free media. Amplification of *nifH* gene segment yielded the product of expected size (420 bp) from DNA template of only one isolate JPA2. Further, considerable acetylene reduction of 38.9 nmol ethylene h^−1^ mg^−1^ protein confirmed the presence of nitrogenase activity.

**Fig. 1:**
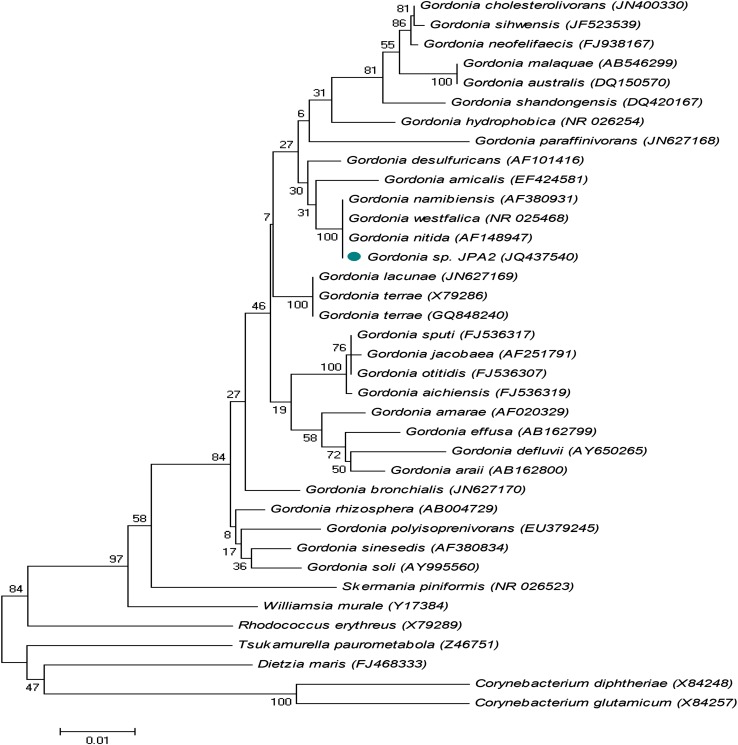
Phylogenetic tree showing the position of strain JPA2 *Gordonia* species and some other related taxa based on 16S rRNA gene sequences. Distances were calculated by neighbor-joining method. Bootstrap values (expressed as percentages of 1,000 replications) are shown at branch points. Genbank accession numbers are given in parentheses. The *scale bar* indicates 0.01 substitutions per nucleotide position

Identification of the strain JPA2 was done on the basis of partial 16S rRNA gene sequencing from Bioserve, Hyderabad (India). Figure 1 represents the phylogenetic position of the detected partial 16S rRNA sequence of JPA2 compared to sequences published in GenBank database. Phylogenetic analyses of the strains based on the NJ method with 1,000 bootstrap sampling resulted in two major clusters and strain JPA2 fell within the cluster comprising the *Gordonia* sp. The bootstrap resampling value for the major cluster comprising distantly related *Gordonia* sp. was 84 % and *Rhodococcus erythreus* (X79289) was used as an outgroup. The identification was further confirmed by morphological and biochemical characterization of the isolate. Colony color of JPA2 was yellowish and cells were Gram +ve cocci as observed in other *Gordonia* sp. Exhibition of biochemical traits such as nitrate reduction, aesculin hydrolysis and starch hydrolysis, assimilation of sugars like glycerol, d- cellobiose and *N*-Acetyl d-glucosamine confirmed it as *Gordonia* sp.

The isolate was able to produce significant amount of IAA (193.27 μg ml^−1^), ammonia (0.085 μg ml^−1^) and positive siderophore production was also detected. Strain JPA2 was found to be salt tolerant as it could tolerate up to 6 % NaCl.

To study the effect of bacterial inoculation, a pot house experiment was conducted and JPA2 was used as an inoculant for pearl millet. JPA2 had more stimulating effect on plant growth and showed significant increase in plant height (51.1, 39.9 and 28.8 %) and plant dry weight (55.9, 36.4 and 35.5 %) over uninoculated control plants at EC 0, 6, 8 dS m^−1^, respectively (Table 1). The higher plant height and shoot dry weight response to inoculant compared to control under different salinity levels clearly showed the beneficial role of this rhizobacterium. Such an improvement might be attributed to N_2_-fixing capacity as well as the ability of this PGPR strain to produce growth-promoting substances. The enhancing effect of seeds treated with bioinoculant on growth and yield of pearl millet has been reported by many researchers. (Latake et al. 2009). Tanawy (2009) isolated bacteria from highly saline soil (EC 23 dS m^−1^) that showed a high ability for promoting plants establishment under adverse saline conditions by producing plant growth promoter substances and fixing a high amount of nitrogen in plant ecosystem. Several studies have also shown that application of PGPR *Azospirillum*, *Bacillus* and *Enterobacter* strains as bioinoculants improves plant growth (Tahir et al. 2013). PGPR *Gordonia* sp. S2RP-17 showed plant growth promotion in Zea mays (Hong et al. 2010). Similar to these reports, our findings also demonstrated that strain JPA2 may be used as a bioinoculant for plant growth promotion.

**Table 1:** Effect of JPA2 on plant height and dry weight of the pearl millet plants

Treatment	EC-0 (dS m^−1^)		EC-6 (dS m^−1^)		EC-8 (dS m^−1^)	
	Plant height (cm)	Dry weight (g/plant)	Plant height (cm)	Dry weight (g/plant)	Plant height (cm)	Dry weight (g/plant)
Control	67.83 ± 2.061	3.70 ± 0.080	59.85 ± 1.507	2.85 ± 0.063	53.88 ± 1.882	2.14 ± 0.069
100 % RDF + JPA2	102.5 ± 2.425***	5.77 ± 0.051***	83.75 ± 3.152**	3.89 ± 0.040***	69.42 ± 0.842**	2.90 ± 0.046***

Data represent the mean ± SEM in each group. Group 1: plant height; Group 2: dry weight

** *P* < 0.01

*** *P* < 0.001

In conclusion, all data in the current work emphasize that the inspected strain JPA2 fixed the nitrogen biologically producing substances of plant growth promoters (IAA, ammonia and siderophores) that enhanced growth and productivity of pearl millet plants when inoculated with this strain, under normal and saline conditions. Also this work proved that the isolated strain JPA2 is saline tolerant (6 % NaCl tolerance) and had the ability to tolerate high soil salinity. Therefore, this strain proved to be potential candidate for the development of bioinoculant for crop plants growing in saline soils.
